# A blood based mitochondrial functional index biomarker for Alzheimer's disease

**DOI:** 10.1002/alz.71061

**Published:** 2025-12-28

**Authors:** Brittany M. Hauger, Riley E. Kemna, Paul J. Kueck, Taylor A. Strope, Casey S. John, Keith P. Smith, Hana D. Mayfield, Ellen Herrold, Keri L. Cox, Rebecca Bothwell, Leonidas Bantis, Russell H. Swerdlow, Jill K. Morris, Heather M. Wilkins

**Affiliations:** ^1^ Department of Neurology University of Kansas Medical Center Kansas City Kansas USA; ^2^ University of Kansas Alzheimer's Disease Research Center Kansas City Kansas USA; ^3^ Department of Biochemistry and Molecular Biology University of Kansas Medical Center Kansas City Kansas USA; ^4^ Department of Biostatistics University of Kansas Medical Center Kansas City Kansas USA; ^5^ Department of Cell Biology and Physiology University of Kansas Medical Center Kansas City Kansas USA

**Keywords:** Alzheimer's disease, amyloid, biomarker, cognition, mitochondria, neurodegeneration, tau

## Abstract

**INTRODUCTION:**

Alzheimer's disease (AD) pathology is complex and involves mitochondrial dysfunction. There are emerging therapies targeting mitochondrial function in clinical trials for AD. This highlights the need for biomarkers that measure mitochondrial function.

**METHODS:**

We determined the utility of a novel blood‐based mitochondrial biomarker, the mitochondrial functional index (MFI), in the context of AD in a pilot study.

**RESULTS:**

In vitro and in vivo models of AD had a reduced MFI. MFI was lower in human AD subjects and *APOE* 𝜀4 carriers. Receiver operating characteristic analysis showed MFI had a higher area under the curve than other plasma biomarkers. The MFI biomarker correlated with the Mini‐Mental State Examination (MMSE) and the Clinical Dementia Rating (CDR) scale.

**DISCUSSION:**

This study highlights the potential utility of MFI as a functional blood‐based mitochondrial biomarker to interrogate energy metabolism. Ongoing studies are examining the relationship of MFI with brain energy metabolism outcomes.

**Highlights:**

- The MFI biomarker is reduced in cell and animal models of AD.
- The MFI biomarker is reduced in human AD subjects and APOE ε4 carriers.
- The MFI biomarker can discriminate between subjects with normal cognition and AD with better performance than other plasma biomarkers.
- The MFI biomarker correlates with cognitive scores.

## 1 BACKGROUND

Alzheimer's disease (AD) pathology begins decades before clinical onset of dementia. Amyloid beta (Aβ) generally accumulates first in cognitively normal individuals, with tau and cognitive abnormalities following.^1^ Metabolic changes are prominent in AD, and mitochondrial function correlates with amyloid and tau measures in cell and animal AD models in addition to human subjects with AD^2^, ^3^, ^4^, ^5^, ^6^, ^7^.

Fluorodeoxyglucose positron emission tomography (FDG PET) comparing AD and cognitively normal individuals reveals lower glucose uptake or metabolism in the brains of AD patients.^8^, ^9^, ^10^, ^11^ Beyond reductions in brain glucose metabolism, mitochondrial dysfunction is observed not only within the brain but also systemically in AD.^12^, ^13^, ^14^, ^15^, ^16^, ^17^, ^18^, ^19^, ^20^, ^21^ More recent genome‐wide association studies (GWASs) identified risk‐associated single nucleotide polymorphisms (SNPs) in genes that function in mitochondrial and metabolic pathways.^22^, ^23^, ^24^, ^25^ Apolipoprotein E (*APOE*), the strongest genetic risk factor for sporadic AD, is central to lipid metabolism and has been found to interact with inherited mitochondrial genes to amplify risk for AD.^25^, ^26^, ^27^ Moreover, *post mortem* AD brain has an overall reduction in the number of intact mitochondria and mitochondrial DNA (mtDNA).^21^, ^28^ Mitochondrial dysfunction plays a role in protein aggregation, inflammation, and cell death – all events observed in AD. Overall, metabolism and mitochondrial dysfunction are strongly associated with AD.

**RESEARCH IN CONTEXT**

1. **Systematic review**: Mitochondrial biomarkers are needed to assess response outcomes in clinical trials for AD. Targeting mitochondrial function and energy metabolism are emerging therapeutic efforts, and without validated mitochondrial biomarkers it is difficult to assess treatment efficacy. Blood‐based mitochondrial biomarkers are an opportunity to measure noninvasive dynamic changes in mitochondrial function.
2. **Interpretation**: The novel MFI biomarker was reduced in models of AD and humans with AD. It outperformed SIMOA‐based plasma biomarkers (Aβ, pTau181, GFAP, and NfL) in discriminating between normal cognition and AD. The MFI biomarker correlated with cognitive testing.
3. **Future directions**: Ongoing studies are examining if the MFI biomarker reflects brain energy metabolism using FDG PET and recent FDA‐approved plasma biomarkers for AD (pTau217 and pTau217/Aβ_42_).

Targeting metabolic and mitochondrial function in clinical trials is an emerging theme for AD. This highlights the importance and need for biomarkers that measure mitochondrial function in a non‐invasive manner. Overall, AD lacks adequate available therapies. Drug and lifestyle intervention development has been difficult due to a lack of therapeutic response biomarkers. Biomarkers are essential for therapeutic response outcomes for AD and to facilitate treatment development efforts.

Measuring mitochondrial function in blood could serve as a powerful tool to understand mitochondrial dynamics in disease and as potential response biomarkers for clinical trials. However, mitochondrial function measures can be complex, noisy, and unreliable. We have published studies using prior blood‐based mitochondrial measures in therapeutic clinical trials for amyotrophic lateral sclerosis (ALS) and AD as secondary response outcomes.^29^, ^30^, ^31^, ^32^, ^33^, ^34^, ^35^ Some of these mitochondrial measures were flow cytometry‐based fluorescent measures of mitochondrial superoxide (MitoSox), mitochondrial membrane potential (tetramethylrhodamine, ethyl ester [TMRE]), and apoptosis. These individual measures had high variability, so we worked to develop an algorithm to overcome these issues and provide a comprehensive measure to assess mitochondrial function. Here, we describe a novel blood‐based mitochondrial biomarker that is measured from peripheral blood mononuclear cells (PBMCs) using fluorophores and flow cytometry. Measures are used in a log‐based algorithm to calculate a mitochondrial functional index (MFI). Here we describe the potential utility of the MFI biomarker in AD in a pilot study.

## 2 METHODS

### 2.1 Human subjects

This study was approved by the Ethics Committee at the University of Kansas Medical Center (FWA00003411). All participants provided written informed consent prior to enrollment in the study. This research was conducted ethically in accordance with the World Medical Association Declaration of Helsinki. We enrolled 20 non‐demented (ND), 20 mild cognitive impairment (MCI), and 19 AD subjects from the University of Kansas AD Research Center (KU ADRC) clinical cohort. Inclusion criteria for this group included individuals diagnosed with AD, MCI, or probable AD or cognitively normal individuals at least 18 years of age and patients who were willing to give informed consent. Exclusion criteria included diagnosis of other neurodegenerative diseases (e.g., Parkinson's disease, ALS), clinically significant history of unstable medical illness (e.g., unstable angina, advanced cancer) over the last 30 days, limited mental capacity such that the patient cannot provide written informed consent or comply with evaluation procedures, history of recent alcohol or drug abuse, or non‐compliance with treatment or other experimental protocols. Mini‐mental State Examination (MMSE) and Clinical Dementia Rating (CDR) data were collected from the KU ADRC cohort. These exams are administered to clinical cohort participants yearly.

We also enrolled 10 healthy volunteers with the following inclusion criteria: no diagnosis of neurodegenerative disease or other significant clinical illness, between 18 and 35 years old, and patients who were willing to give informed consent. Exclusion criteria included diagnosis of neurodegenerative diseases (e.g., AD, dementia, Parkinson's disease, ALS), clinically significant history of unstable medical illness (e.g., unstable angina, advanced cancer) over the last 30 days, the history of recent alcohol or drug abuse or non‐compliance with treatment or other experimental protocols, persons with excessively low blood pressure (systolic blood pressure < 85, diastolic blood pressure < 55), excessive bradycardia (pulse < 55) or tachycardia (pulse > 100), persons who have had adverse events such as fainting or excessive bleeding during a past phlebotomy, persons who had a greater than 100 cc phlebotomy within the past 2 weeks prior to the study, or who are anticipated to undergo more than 100 cc of phlebotomy during the next 2 weeks of the study. Human subject demographics for ND, AD, and MCI groups are represented in Table 1. Healthy volunteers had an average age of 28.6 years with a standard deviation of 2.7 years, all White/non‐Hispanic, five male and five female subjects.

**TABLE 1:** Demographics.

	ND	MCI	AD
Age (SD)	77.8 (5.6)	78.1 (8.4)	77.9 (8.1)
Sex (M/F)	8/12	10/10	12/7
APOE ε4 (*n*)	5	10	9
CDR (SD)	0	0.5	0.86 (0.23)
MMSE (SD)	28.3 (1.5)	26.5 (3.1)	20.1 (5.1)
*n*	20	20	19
**Demographics**	**ND**	**MCI**	**AD**
White	19	19	17
Black American		1	
Unknown			1
Asian	1		1
**Medication use**	**ND**	**MCI**	**AD**
Statins	15	16	9
Supplements	14	17	16
Blood pressure	5	6	2
Memantine	0	2	10
Donepezil	0	8	7
SSRIs	0	1	2
Hormones/thyroid	6	1	3
Diabetes	3	4	1

Abbreviations: AD, Alzheimer's disease; APOE, apolipoprotein E; CDR, Clinical Dementia Rating; MMSE, Mini‐Mental State Examination; MCI, mild cognitive impairment; ND, non‐demented; SD, standard deviation.

### 2.2 Study visits

For ND, MCI, and AD subjects, only one study visit was completed. After subjects were consented, a phlebotomy was performed where three acid citrate dextrose (ACD) tubes (8.5 mL each) and one EDTA tube (9 mL) for a total phlebotomy volume of ∼35 mL was collected.

For healthy volunteers, the study involved four visits. For visit 1, after informed consent, subjects received a questionnaire asking about medication/supplement use, alcohol use, and smoking history. Subjects underwent a fasting phlebotomy in three ACD tubes (8.5 mL each) and one EDTA tube (10 mL) for a total volume of ∼36 mL. For visit 2, subjects underwent a non‐fasting phlebotomy of three ACD tubes (8.5 mL each) and one EDTA tube (10 mL) for a total volume of ∼36 mL. Subjects completed a dietary recall questionnaire. For visit 3, subjects underwent a fasting phlebotomy in three ACD tubes (8.5 mL each), three EDTA tubes (9 mL each), and three heparin tubes (10 mL each) for a total volume of ∼83 mL. For visit 4, subjects underwent a fasting phlebotomy in 12 ACD (8.5 mL each) tubes for a total volume of ∼102 mL.

### 2.3 Blood processing

PBMCs were isolated using Accuspin tubes, Histopaque 1077, and differential centrifugation as previously described.^30^, ^31^, ^34^ For mitochondrial biomarkers in the ND, MCI, and AD groups, blood was collected in ACD tubes and processed/measured within 30 h of blood draw. For visits 1, 2, and 3 of the healthy control group, blood was collected in varying anticoagulant types (EDTA, ACD, and heparin), and blood was processed/measured for mitochondrial biomarkers the same day. For visit 4 of the healthy control group, blood was collected in ACD tubes and processed/measured for mitochondrial biomarkers over varying times (less than 24, 48, 72, and 96 h) following phlebotomy. For A/T/N analyses, blood was collected in EDTA tubes and spun down at 500 x g for 15 min. Plasma was collected, aliquoted, and frozen until measures were completed.

### 2.4 Induced pluripotent stem cell (iPSC) source and reprogramming

iPSCs were reprogrammed from dura fibroblasts obtained at the KU ADRC or purchased from WiCell. KU ADRC fibroblast donors were members of the clinical cohort, who consented to donation upon death, and approval from an Ethical Standards Committee to conduct this study was received. The studies involving human participants were reviewed and approved by the University of Kansas Medical Center Institutional Review Board. Banked tissue is de‐identified by the KU ADRC Neuropathology Core to eliminate identifying information. Reprogramming was completed using the Sendai Virus, CytoTune‐iPS 2.0 Sendai Reprogramming Kit from Thermo Fisher Scientific. iPSCs were age, sex, and diagnosis matched (*n* = 4 per diagnosis). For iPSCs derived from the KU ADRC cohort, ND or AD were diagnosed at autopsy neuropathological examination as outlined in the National Alzheimer's Coordinating Center Neuropathology Coding Guidebook.^36^

### 2.5 iPSC neural progenitor cell differentiation

iPSCs were differentiated into neural progenitor cells (NPCs) using STEMDiff Neural Induction Medium (NIM). iPSCs were placed into a single‐cell suspension in NIM with SMADi/ROCKi (SMAD inhibitor and ROCK inhibitor) in an AggreWell 800 plate. Embryoid bodies were cultured in the AggreWell plate for 5 days with NIM/SMADi partial medium changes daily. Embryoid bodies were then plated onto Matrigel‐coated plates and fed daily with NIM/SMADi medium until day 12 to allow neural rosette formation. Neural rosettes were selected using Neural Rosette Selection Reagent (StemCell Tech) and plated onto Matrigel‐coated dishes with NIM/SMADi. Medium was changed daily for 7 days, after which NPCs were cryopreserved and split into defined neural progenitor medium (StemCell Tech).

### 2.6 iPSC forebrain neuronal differentiation

NPCs were plated onto Poly‐L‐Ornithine (PLO)/laminin‐coated dishes in neural progenitor medium. The following day the media was changed to StemDiff Forebrain Neural Differentiation Medium (StemCell Tech). Medium was changed daily for 7 days,^37^, ^38^ following which, cells were plated onto PLO/laminin‐coated dishes in defined Brain Phys Medium (with N2A, SM1, BDNF, GDNF, cAMP, and ascorbic acid) for neuronal maturation. Neurons were matured for 7 to 10 days and used for downstream experiments.

### 2.7 iPSC astrocyte differentiation

NPCs were plated onto Matrigel‐coated dishes in neural progenitor medium. The following day cells were placed in astrocyte differentiation medium consisting of DMEM (high glucose, with glutamine, no pyruvate), B27, 1% fetal bovine serum, glutamine, bFGF, CNTF, BMP8, Activin A, heregulin 1b, and IGF1. Medium was changed every other day, and cells were passaged as needed.^39^ After 30 days or approximately five or six passages, astrocytes were used in downstream experiments.

### 2.8 5xFAD mice

All animals were housed in a temperature‐ (∼25°C) and light‐controlled (12:12 h light‐dark) room with free access to food and water. Mice were euthanized in the morning between 8:00 and 10:00 a.m., with CO2 and decapitation, where trunk blood was collected in ACD solution. PBMCs were isolated using histopaque 1077 and differential centrifugation. The Institutional Animal Care and Use Committee (A3237‐01) approved the animal protocols at the University of Kansas Medical Center (*n* = 3 per group [two males, one female] at ∼8 weeks of age.

### 2.9 Mitochondrial functional index

Approximately two million PBMCs were stained with Annexin V+MitoTracker, MitoSox+ Hoechst, and TMRE+ Hoechst as previously described.^30^, ^31^ Briefly, cells were incubated with 40 nM MitoTracker, 5 µM MitoSox/10 ng Hoechst, and 200 nM TMRE/10 ng Hoechst (in separate tubes) for 30 min at 37°C/5% CO_2_ in Hanks Balanced Salt Solution (HBSS with Ca2+/Mg2+). MitoTracker cells are washed and then stained with Annexin V in binding buffer, then diluted for flow cytometry analysis. MitoSox and TMRE‐stained cells are washed with HBSS and diluted for flow cytometry analysis, where 10,000 cells per tube were analyzed. All values were normalized to Hoechst signal. Fluorescent measures were completed within 30 h of blood draw. The MFI algorithm – MFI = log [(MitoTracker x TMRE)/(MitoSox x Annexin V)] – provides an overall picture of mitochondrial health and function. The MFI biomarker is listed in utility patent no. 63/824,391 *(MFI)* as of June 16, 2025.

### 2.10 A/T/N measures

Whole blood was collected via venipuncture into vacutainer tubes containing EDTA as an anticoagulant. Blood was centrifuged at 1800 × g for 10 min to separate plasma, which was aliquoted and stored at −80°C prior to analyses. Fluid biomarkers were analyzed on Quanterix Simoa HD‐X (Neuro 4 Plex E [N4PE; Aβ40, Aβ42, neurofilament light [NfL], glial fibrillary acidic protein [GFAP], and pTau181 versions 2.0 and 2.1 Advantage) per manufacturer's instructions using appropriate calibrators and controls. Because samples were analyzed using two versions of the pTau181 assay, a conversion factor published by Quanterix was employed to adjust values for samples analyzed on pTau181 version 2.1.

### 2.11 APOE genotyping

A SNP allelic discrimination assay was used to determine APOE genotypes. This involved adding 5 ul blood to a Taqman Sample‐to‐SNP kit (Thermo Fisher Scientific). Taqman probes to the two APOE‐defining SNPs, rs429358 (C_3084793_20) and rs7412 (C‐_904973_10) (Thermo Fisher Scientific), were used to identify APOE ε2, ε3, and ε4 alleles.

### 2.12 Data analysis

All experiments were completed on at least three different sets of differentiated cells or mice. Data were summarized by means and standard deviations or errors. To compare means between three or more groups, we used one‐way ANOVA followed by Fisher's least significant difference (LSD) post hoc test. To compare means between two groups, we used two‐way, unpaired Student's *t* tests. Receiver operating characteristic (ROC) analysis was also conducted. We provide empirical ROC estimates, along with the corresponding empirical area under the curve (AUC) estimates. For inferences and comparisons regarding the ROC analysis, we utilized the bootstrap with 1000 bootstrap iterations. The biomarker cutoffs reported are based on the maximization of the empirical Youden Index. The ROC analysis was conducted using MATLAB (Mathworks). Correlation analyses used Pearson's test. Statistical tests were performed using Prism/GraphPad. *P* values less than 0.05 were considered statistically significant.

## 3 RESULTS

**FIGURE 1:**
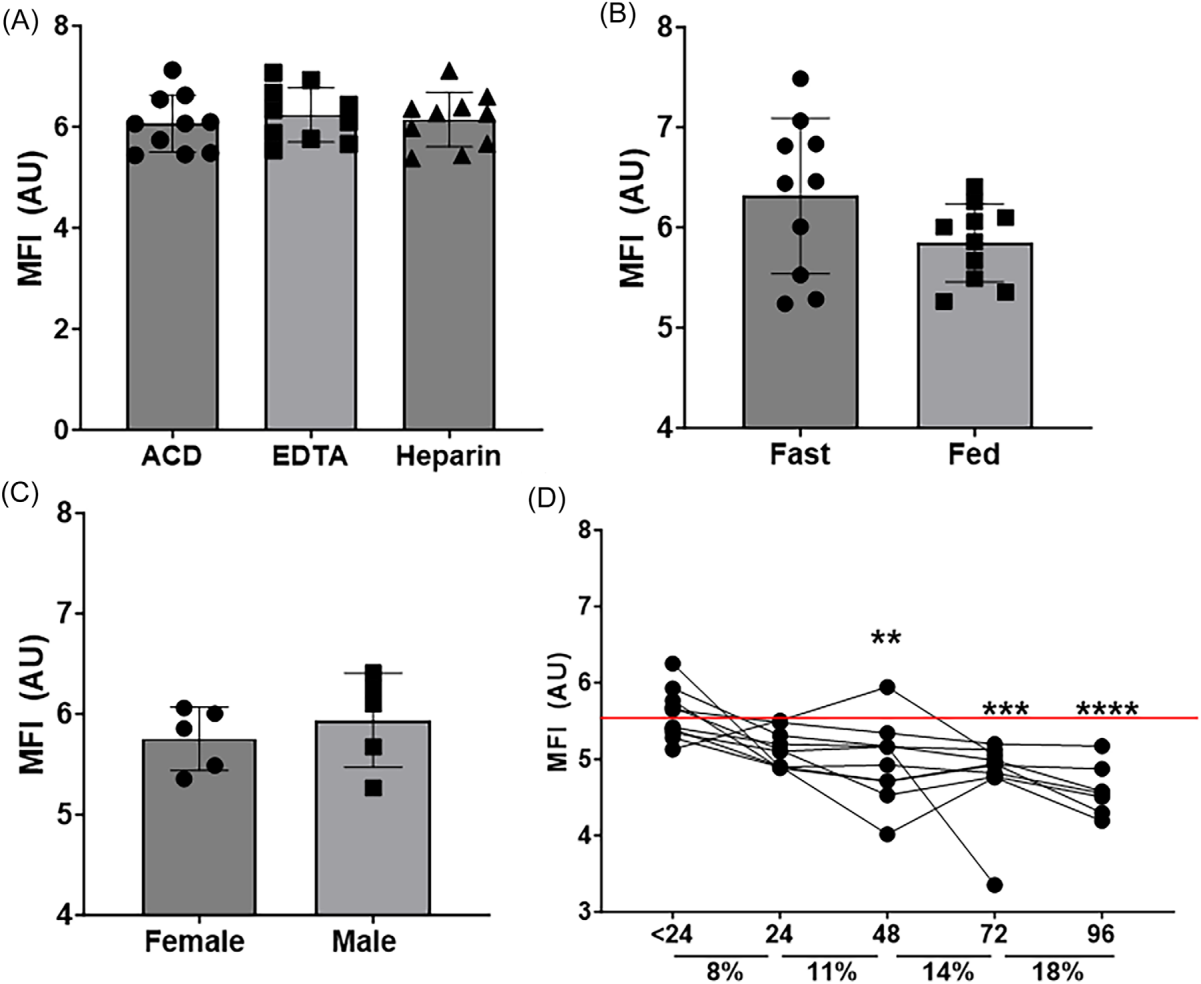
Mitochondrial functional index (MFI) biomarker development. (A) MFI measures with different anticoagulant types including ACD, EDTA, and heparin; *n* = 10. (B) MFI in fasting versus fed state, *n* = 10. (C) MFI by sex; *n* = 5 each. (D) MFI following different blood processing times *y*‐axis indicates hours since blood draw and percentage reduction in biomarker following preceding time point. Mean with standard deviation.

The MFI biomarker combines mitochondrial reporter dyes for TMRE, MitoSox, mitochondrial mass (MitoTracker), and apoptosis (Annexin V) in an algorithm to assess overall mitochondrial function. A higher MFI value is associated with higher mitochondrial function. In human iPSC‐derived neurons from a healthy donor, treatment with rotenone and antimycin A significantly reduced the MFI biomarker (Figure 1A). Rotenone and antimycin A are complex I and complex III inhibitors, respectively. We next examined the levels of the MFI biomarker in iPSC‐derived neurons and astrocytes from ND and sporadic AD subjects. The MFI biomarker was significantly reduced in both neurons and astrocytes derived from AD subject iPSCs (Figure 1B,C). To further examine if the MFI biomarker was altered in models of AD, we examined it in PBMCs from 5xFAD mice at 8 weeks of age. There was a significant reduction in MFI in the 5xFAD mice (Figure 1D).

**FIGURE 2:**
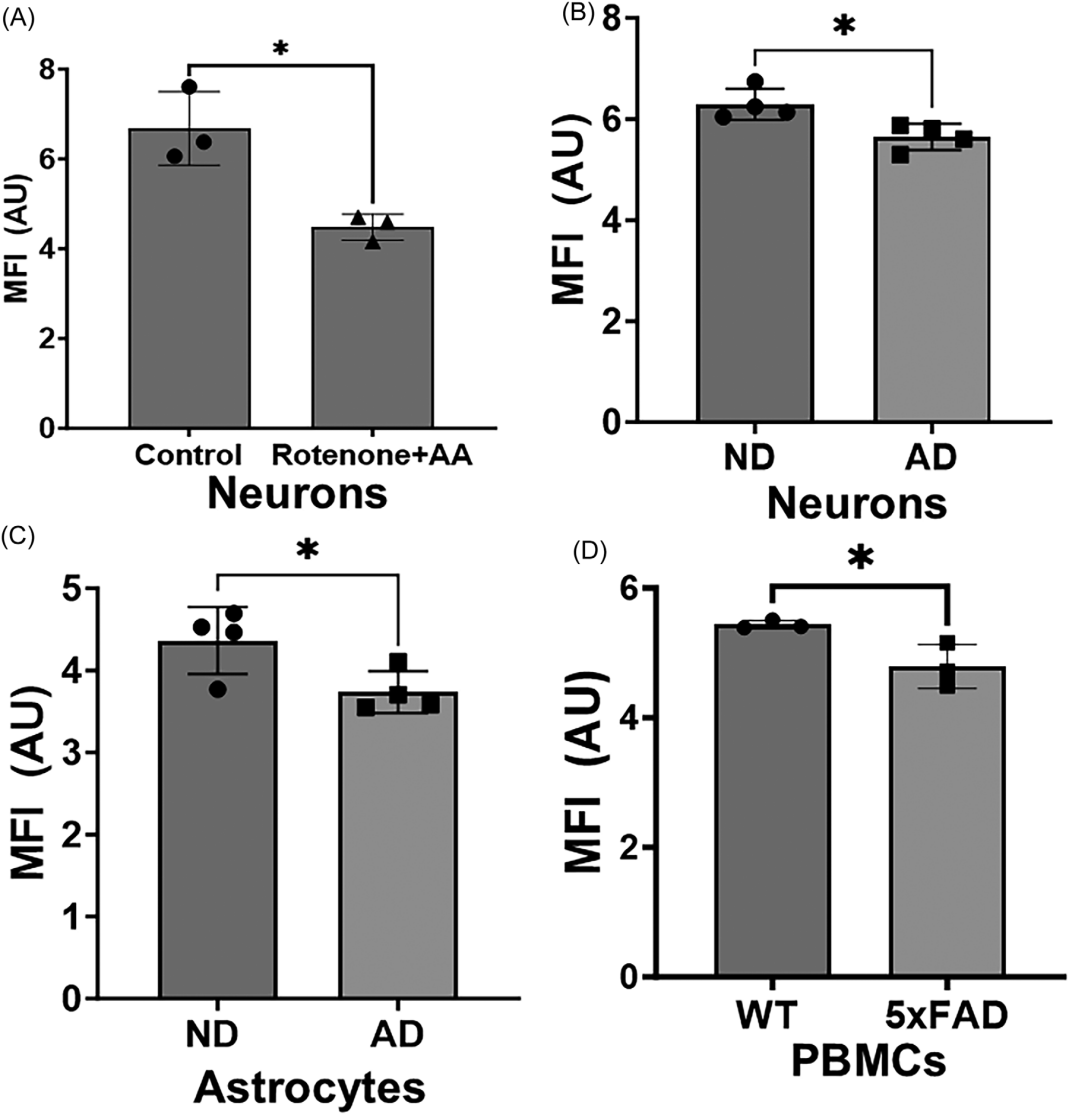
Mitochondrial functional index (MFI) biomarker in Alzheimer's disease models. (A) MFI in iPSC derived neurons treated with mitochondrial toxins (1 µM each rotenone and antimycin A, 1 h); *n* = 3 per group. (B) MFI measure in induced pluripotent stem cell (iPSC)‐derived neurons; *n* = 4 per group. (C) MFI measure in iPSC‐derived astrocytes; *n* = 4 per group. (D) MFI measure in 5xFAD mice (two males, one female per group) at age 8 weeks from peripheral blood mononuclear cells; *n* = 3 per group. Mean with standard deviation.

To establish standardized protocols and stability for the MFI biomarker in human blood, we enrolled 10 healthy (18 to 35 years old, five male/ five female) individuals. No difference in MFI biomarker levels was observed between ACD, EDTA, or heparin anticoagulants (Figure 2A). We observed no difference in MFI biomarker level between fasting or fed blood draws (Figure 2B) or between male and female participants (Figure 2C). The stability of the MFI biomarker showed significant reductions in levels between 24 and 48 h following blood collection in ACD tubes (Figure 2D). With each 24‐h period there was an approximate 8% to 18% reduction in MFI biomarker levels. Based on these findings, all biomarker processing was completed within 30 h of blood collection for this study.

**FIGURE 3:**
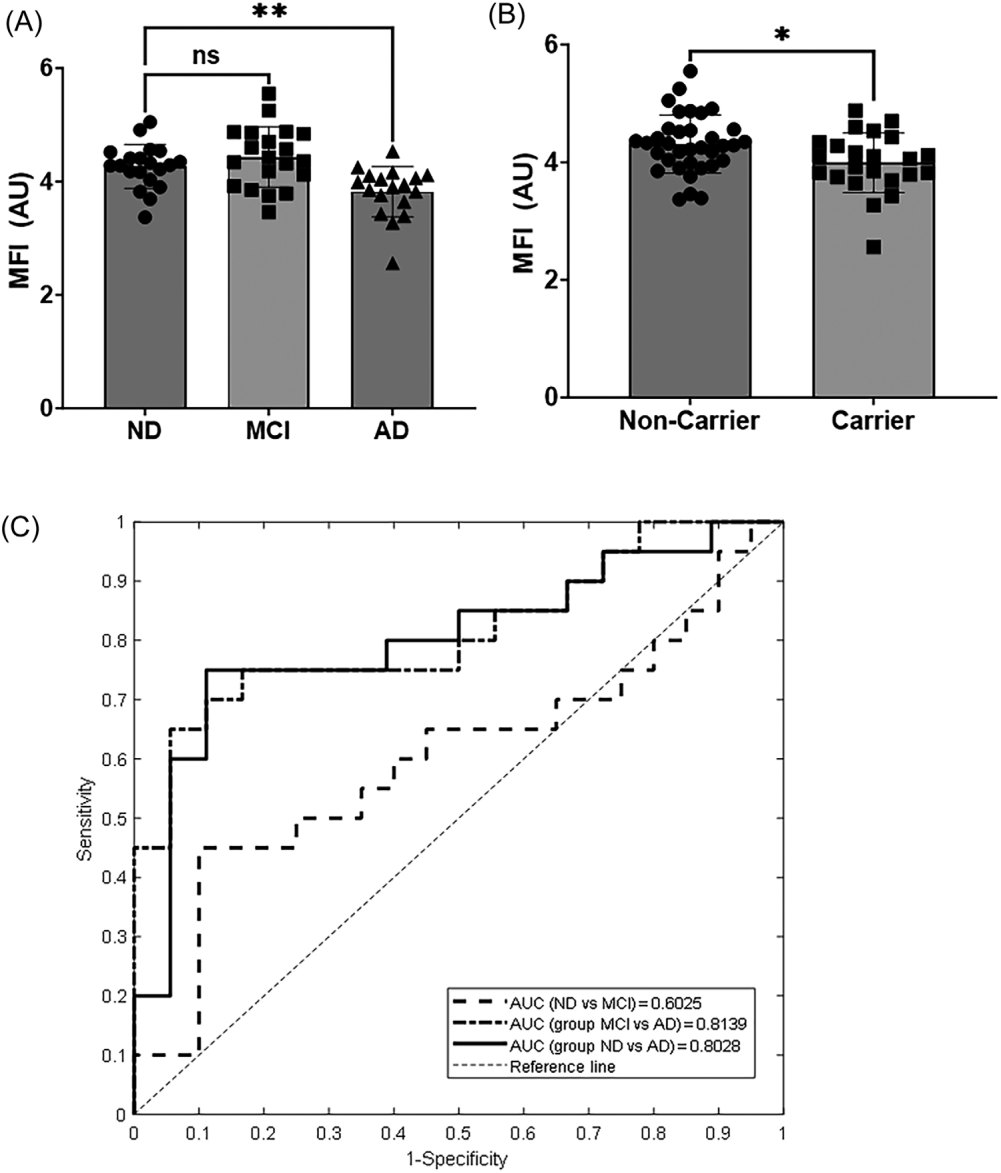
MFI biomarker in Alzheimer's disease (AD). (A) Mitochondrial functional index (MFI) measures in non‐demented (ND), mild cognitive impairment (MCI), and AD. *n* = 20 for ND and MCI, *n* = 19 for AD. (B) MFI by *APOE4* carrier status *n* = 34 non‐carriers, *n* = 23 carriers. (C) Area under the curve analysis discrimination between ND/AD, ND/MCI, or MCI/AD. Mean standard deviation.

We next examined the MFI biomarker in ND, MCI, and AD subjects. A significant reduction in MFI biomarker was observed in the AD group when compared to the ND group (Figure 3A). The MFI biomarker was also lower in individuals that carry an *APOE* 𝜀4 allele regardless of diagnosis (Figure 3B). Examination of the ROC analysis of the MFI biomarker showed an AUC of 0.8028 between ND and AD, 0.6025 between ND and MCI, and 0.8139 between MCI and AD (Table 2, Figure 3C). The MFI is composed of four biomarkers in its algorithm, and each individual biomarker AUC and cutoff for discrimination between diagnoses are shown in Table 2. The AUC ranged from 0.5526 to 0.622 for individual biomarkers compared to 0.8028 for the MFI algorithm. Plasma A/T/N biomarkers showed significant differences between ND and AD groups for pTau181, NfL, and GFAP (Table 3). The MCI group had significantly increased plasma pTau181 values compared to the ND group (Table 3). Cutoffs for plasma biomarker measures are listed in Table 3. When comparing the MFI biomarker performance to other plasma ATN biomarkers, the MFI showed a similar AUC for discrimination between ND and AD compared to pTau181, GFAP, NfL, and Aβ_42/40_ (Table 4). For discrimination between MCI and AD, MFI performed better than pTau181, GFAP, NfL, and Aβ_42/40_ (Table 4).

**TABLE 2:** Mitochondrial Biomarker.

ROC	AUC	SE	CI_lower	CI_upper	*P* value	Sensitivity at 95% specificity	Specificity at 95% sensitivity	Cutoff	Specificity at cutoff	Sensitivity at cutoff
Individual mitochondrial biomarker ROC (ND vs AD)										
TMRE	0.6222	0.0938	0.4384	0.8061	0.0963	0.05	0.1111	0.2745	0.6112	0.6500
MitoSox	0.5658	0.094	0.3816	0.75	0.242	0.1053	0.05	0.01405	0.5500	0.6316
Annexin V	0.5961	0.0895	0.4206	0.7715	0.1417	0.1053	0.05	36.500	0.8000	0.4210
MitoTracker	0.5526	0.0943	0.3678	0.7375	0.2884	0.1	0	65128	0.9474	0.2500
MFI composite biomarker ROC										
MFI (ND vs AD)	0.8028	0.0729	0.6599	0.9457	0	0.2	0.1111	4.1689	0.8889	0.7500
MFI (ND vs MCI)	0.6025	0.0932	0.4198	0.7852	0.1358	0.1	0.05	4.5748	0.9000	0.4500
MFI (MCI vs AD)	0.8139	0.069	0.6787	0.9491	0	0.45	0.2222	4.3158	0.9444	0.6500

Abbreviations: AD, Alzheimer's disease; AUC, area under the curve; CI, confidence interval; MCI, mild cognitive impairment; MitoSox, mitochondrial superoxide; ND, non‐demented; ROC, receiver operating characteristic; SE, standard error; TMRE, tetramethylrhodamine, ethyl ester.

**TABLE 3:** Plasma biomarkers (mean ± SD).

Group	Aβ_42_/Aβ_40_	pTau181	GFAP	NfL
ND	0.089 (0.014)	2.42 (0.91)	158.2 (66)	18 (6.5)
MCI	0.0819 (0.0168)	3.89 (2)*	170.4 (80)	18.8 (7.4)
AD	0.0847 (0.0117)	3.80 (1.5)*	228.9 (101)*	24.1 (8.9)*
ND versus AD cutoffs				
Cutoff	0.088	3.2577	139.8744	20.0479
Specificity at cutoff	0.7895	0.8421	0.55	0.7
Sensitivity at cutoff	0.55	0.6875	0.8889	0.7222
ND versus MCI cutoffs				
Cutoff	0.0856	3.6171	182.9405	23.9114
Specificity at cutoff	0.7	0.9474	0.7	0.85
Sensitivity at cutoff	0.65	0.5625	0.5263	0.3684
MCI versus AD cutoffs				
Cutoff	0.0757	3.8323	134.18199	16.6687
Specificity at cutoff	0.4	0.5625	0.3684	0.579
Sensitivity at cutoff	0.8421	0.5625	0.9444	0.8333

Abbreviations: Aβ, amyloid beta; AD, Alzheimer's disease; GFAP, glial fibrillary acidic protein; MCI, mild cognitive impairment; ND, non‐demented; NfL, neurofilament light; pTau181, tau phosphorylated at threonine 181.

**TABLE 4:** Biomarker AUC comparisons.

ND/AD	Biomarker	AUC	SE	CI_lower	CI_upper	*P* value‐1t	Sensitivity at 95% specificity	Specificity at 95% sensitivity	MFI comparison *p* value‐1t
	pTau181	0.7796	0.08	0.62	0.94	0.00	0.50	0.11	0.35
	GFAP	0.7194	0.08	0.56	0.88	0.00	0.39	0.05	0.26
	NfL	0.7194	0.08	0.56	0.88	0.00	0.28	0.10	0.22
	Aβ_42_/Aβ_40_	0.6658	0.09	0.49	0.84	0.03	0.00	0.00	0.10
	MFI	0.8028	0.07	0.66	0.95	0.00	0.20	0.11	‐
**ND/MCI**	**Biomarker**	**AUC**	**SE**	**CI_lower**	**CI_upper**	***P* value‐1t**	**Sensitivity at 95% specificity**	**Specificity at 95% sensitivity**	**MFI comparison *p* value‐1t**
	pTau 181	0.7105	0.09	0.53	0.89	0.01	0.44	0.00	0.37
	GFAP	0.5737	0.10	0.38	0.76	0.22	0.21	0.00	0.47
	NfL	0.5368	0.10	0.35	0.73	0.35	0.21	0.00	0.36
	Aβ_42_/Aβ_40_	0.6700	0.09	0.50	0.84	0.03	0.05	0.05	0.0144*
	MFI	0.6025	0.09	0.42	0.79	0.14	0.10	0.05	‐
**MCI/AD**	**Biomarker**	**AUC**	**SE**	**CI_lower**	**CI_upper**	***P* value‐1t**	**Sensitivity at 95% specificity**	**Specificity at 95% sensitivity**	**MFI comparison *p* value‐1t**
	pTau181	0.5078	0.11	0.30	0.72	0.47	0.00	0.13	0.0014*
	GFAP	0.5965	0.10	0.40	0.79	0.16	0.00	0.11	0.0279*
	NfL	0.6462	0.09	0.46	0.83	0.06	0.00	0.05	0.06
	Aβ_42_/Aβ_40_	0.5632	0.10	0.38	0.75	0.25	0.05	0.15	0.0120*
	MFI	0.8139	0.07	0.68	0.95	0.00	0.45	0.22	‐

Abbreviations: Aβ, amyloid beta; AD, Alzheimer's disease; AUC, area under the curve; CI, confidence interval; GFAP, glial fibrillary acidic protein; MCI, mild cognitive impairment; MFI, mitochondrial functional index; NfL, neurofilament light; pTau181, tau phosphorylated at threonine 181; SE, standard error

**FIGURE 4:**
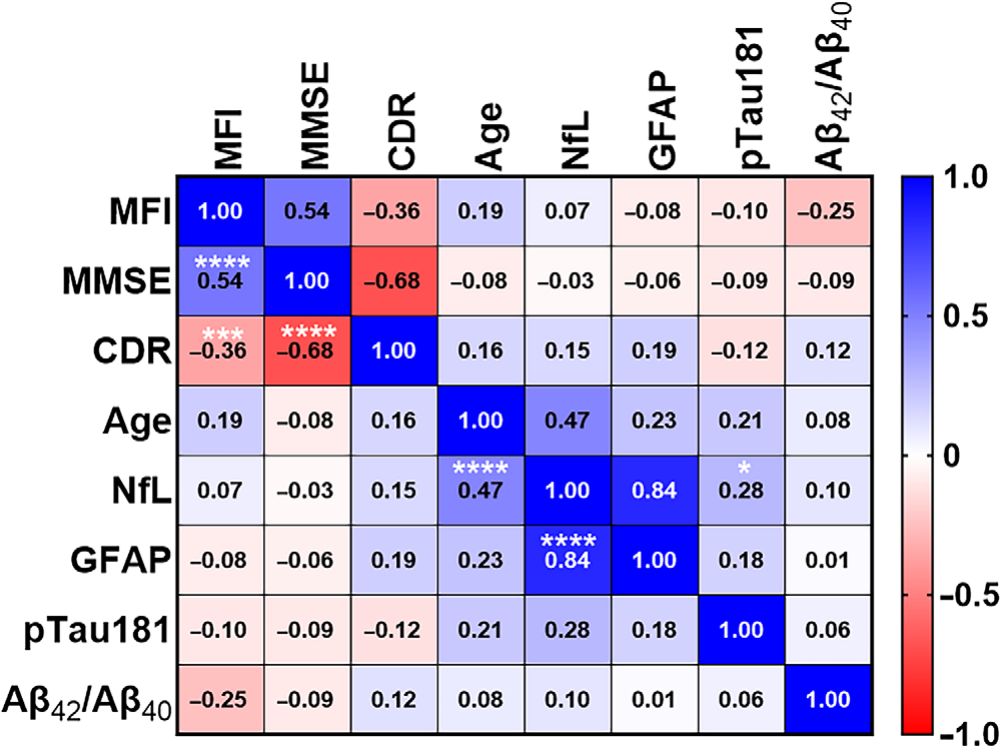
MFI correlation with other biomarkers. Correlation matrix between MFI and all other biomarkers; numbers show Pearson's *R*. Significant correlations were observed between MMSE/MFI *p* = 0.00015, CDR/MFI *p* = 0.005, pTau181/NfL *p* = 0.05, age/NfL, *p* = 0.00019, GFAP/NfL *p* = 1.73 × 10^−16^. CDR, Clinical Dementia Rating; GFAP, glial fibrillary acidic protein; MFI, mitochondrial functional index; MMSE, Mini‐Mental State Examination; NfL, neurofilament light; pTau 181, tau phosphorylated at threonine 181.

The MFI biomarker significantly correlated with MMSE and CDR (Figure 4). MMSE showed a significant correlation with CDR as well. Plasma NfL correlated with age, plasma GFAP, and plasma pTau181 (Figure 4).

Finally, we examined if commonly used therapies for AD that have been implicated in modulating mitochondrial function affect the MFI biomarker. We saw no evidence that donepezil affected the MFI biomarker in the AD group (data not shown).

## 4 DISCUSSION

We sought to develop a blood‐based biomarker to measure mitochondrial function. As mitochondrial function is dynamic and encompasses many biological processes, we chose four variables to calculate a novel algorithm. We focused on assays that use flow cytometry‐based methods in consideration of accessibility and affordability. The variables included in the MFI biomarker include TMRE, MitoSox, MitoTracker, and Annexin V. TMRE is a measure of mitochondrial membrane potential and increases or decreases in TMRE, affect adenosine triphosphate production and redox balance.^40^ MitoSox is a measure of mitochondrial superoxide, a direct measure of oxidative stress and redox within mitochondria.^41^ MitoTracker levels indicate mitochondrial mass, which can influence overall energy production and homeostasis.^42^ Annexin V is an apoptotic marker, a process that mitochondria are integral to.^43^ Each of these facets of mitochondrial function is altered in models of AD.^2^, ^5^, ^44^

Current blood‐based biomarkers for AD focus on amyloid, tau, and neurodegeneration (A/T/N) measures in plasma. Amyloid and tau levels are relatively straightforward; however, measuring neurodegeneration in plasma involves the use of surrogates of neuronal and glial brain status, which include NfL and GFAP. Prior studies reported that those with AD have elevated plasma phosphorylated tau (pTau181 or pTau217), reduced Aβ_42_/Aβ_40_ ratios, increased NfL, and increased GFAP.^45^, ^46^, ^47^, ^48^, ^49^, ^50^, ^51^ The MFI biomarker significantly correlated with NfL but no other plasma biomarkers measured in this study. As expected, correlations were observed between GFAP and NfL in addition to NfL and age. The correlation between MFI and NfL is important, as mitochondrial function is highly correlative of neuronal dysfunction and neurodegeneration.

In this study, the MFI biomarker performed better for discrimination between MCI and AD subjects compared with other plasma A/T/N biomarkers. While prior studies showed better AUCs for pTau181 and Aβ ratios in plasma, this could have been due to the size of our study or varying diagnostic criteria. Our blood‐based MFI biomarker performed better than plasma pTau181, GFAP, and Aβ_42_/Aβ_40_ for discrimination between MCI and AD subjects in this study and cohort. Furthermore, the MFI biomarker performed as well as plasma pTau181, GFAP, NfL, and Aβ_42_/Aβ_40_ for discrimination between ND and AD subjects. These data suggest that mitochondrial biomarkers could be used in combination with plasma A/T/N biomarkers to increase sensitivity and specificity in diagnostic and prognostic outcomes for AD. Future studies will address this possibility.

Our overall goal was to develop and test the utility of a blood‐based mitochondrial biomarker that could be used as a response biomarker in future clinical trials. The MFI biomarker can detect mitochondrial dysfunction in human iPSC‐derived neurons treated with known mitochondrial toxins, iPSC‐derived neuron and astrocyte models of sporadic AD, 5xFAD mice, and human AD subjects. While the MFI biomarker did not discriminate between ND and MCI subjects, this is not surprising or concerning. Those with MCI often convert back to ND or eventually convert to AD, so this reflects a heterogeneous population with some MCI. The MFI did correlate with MMSE and CDR cognitive scores. Further studies are needed to interrogate the mechanisms of these correlations and understand if the MFI has any prognostic or predictive utility in cognition and AD. Caution should be exercised with the discriminatory ability of the MFI biomarker between diagnostic states in AD given the small sample size of the current study.

We showed that the MFI biomarker was relatively stable (∼30 h in ACD tubes at room temperature), which allows for overnight shipments of blood to central labs if needed. This is not dissimilar to other labs that require fresh blood for testing in commercialized central labs. However, the MFI biomarker requires additional clinical validation and development before implementation in clinical settings. A benefit of using a flow cytometry‐based assay is that it is highly conducive to the development of standalone high‐throughput instrumentation to measure the MFI; but this requires further engineering and cost/risk analyses.

Other considerations and questions include whether a systemic mitochondrial biomarker reflects brain metabolism. Studies of AD reflect systemic mitochondrial dysfunction observed in blood cells (platelets, PBMCs), skin cells, and muscle; as such, it is not clear whether a blood‐based mitochondrial biomarker is required to reflect brain metabolism for utility.^34^, ^52^, ^53^, ^54^ Improving systemic mitochondrial function is likely to have beneficial effects on brain outcomes, but this is to be determined.

Prior studies have shown significant relationships between blood‐based mitochondrial outcomes and brain metabolism. Blood mtDNA copy number significantly correlates with brain glucose metabolism (FDG PET) and amyloid PET outcomes.^55^ An additional blood‐based mitochondrial measure, platelet cytochrome oxidase (COX) Vmax, significantly associated with FDG PET outcomes.^56^ Based on these studies, it is feasible that blood‐based mitochondrial biomarkers could reflect altered brain metabolism, and this is the focus of ongoing studies.

To attempt to answer the question of the relationships between blood‐based mitochondrial biomarkers and brain metabolism, we have a current ongoing study examining the MFI biomarker, brain FDG PET, brain MRI structure/volume, and newly US Food and Drug Administration‐approved plasma A/T/N biomarkers (ptau217 and ptau217/Aβ_42_) within the same subjects. A limitation of the current study is the small sample size, which highlights the need for further clinical validation of the MFI biomarker in addition to understanding how it performs with and against other plasma biomarkers and neuroimaging.

With further clinical validation and intra‐lab standard operating procedures, we believe the MFI and other potential blood‐based mitochondrial biomarkers could be leveraged in therapeutic clinical trials as response outcomes. This is especially critical for AD and related dementias given the increasing interest in therapies targeting metabolism. It is currently not feasible to know whether a therapy fails in a clinical trial because it fails to engage its intended target (mitochondria/metabolism) or if it fails because the intended target is not disease modifying. The only way to overcome this is through response biomarker development specific to therapeutic targets, like mitochondrial function.

## CONFLICT OF INTEREST STATEMENT

The MFI biomarker is listed in utility patent no. 63/824,391 (mitochondrial functional index) as of June 16, 2025, for Russell Swerdlow and Heather Wilkins. All other authors have nothing to disclose. Author disclosures are available in the Supporting Information.

## CONSENT STATEMENT

All human subjects provided informed consent for this study.

## Supporting information



Supporting Information
